# Parental Decision-Making Process in Termination of Pregnancy for Congenital Heart Disease: From the Multidisciplinary Perinatal Council of a Turkish Tertiary Center

**DOI:** 10.3390/diagnostics16162624

**Published:** 2026-08-19

**Authors:** Abdulmecit Oktem, Mukremin Ceylan, Hakan Golbasi

**Affiliations:** Department of Perinatology, Izmir City Hospital, Izmir 35540, Turkey; mukreminceylan68@gmail.com (M.C.); drhkngolbasi@gmail.com (H.G.)

**Keywords:** congenital heart defects, prenatal diagnosis, abortion, genetic testing, decision making

## Abstract

**Background/Objectives:** Termination of pregnancy (TOP) following a prenatal diagnosis of congenital heart disease (CHD) is a complex decision shaped by clinical, ethical, and psychosocial factors. This study evaluated the clinical factors associated with parental acceptance of TOP after prenatal diagnosis of CHD in a multidisciplinary counselling setting. **Methods:** This retrospective cohort study was conducted at a tertiary referral center where fetal CHD cases are evaluated in a multidisciplinary perinatology council. Pregnancies with a prenatal CHD diagnosis for which TOP was medically recommended between December 2023 and February 2026 were included. Maternal, fetal, and genetic data were analyzed. Cardiac severity was classified using the fetal cardiovascular disease severity scale. Because the cohort included only 62 pregnancies, a simplified multivariable logistic regression model with three clinically prespecified variables (four parameters: gestational age, cardiac severity, and genetic-evaluation status) was used to identify variables associated with termination acceptance. **Results:** Sixty-two pregnancies were included; 41 (66.1%) accepted termination and 21 (33.9%) continued the pregnancy. Maternal characteristics and gestational age at diagnosis were similar between groups. In the multivariable model, genetic-evaluation status was the only variable independently associated with termination acceptance. Compared with cases without genetic testing, both major chromosomal/pathogenic anomalies (aOR 4.97; 95% CI 1.29–19.11; *p* = 0.020) and a normal genetic-evaluation status (aOR 4.71; 95% CI 1.07–20.77; *p* = 0.041) were associated with higher termination acceptance. Cardiac severity and gestational age were not independently associated with parental decision. **Conclusions:** Genetic-evaluation status was associated with parental acceptance of termination after prenatal CHD diagnosis in a multidisciplinary counselling setting; given the small sample size and the selected, council-recommended cohort studied, this association should be interpreted as hypothesis-generating rather than as evidence of a causal or independent predictive effect.

## 1. Introduction

A prenatal diagnosis of congenital heart disease (CHD) presents one of the most challenging situations in fetal medicine. Beyond the technical aspects of cardiac anatomy and surgical prognosis, such a diagnosis places families in a complex decision-making process that involves medical, emotional, and ethical considerations [1]. When termination of pregnancy (TOP) is medically recommended, parents must make a time-sensitive decision while considering the expected prognosis and its possible impact on their family and future [2].

CHD is not limited to a structural cardiac defect alone. It may be associated with genetic syndromes, extracardiac anomalies, and potential long-term neurodevelopmental challenges [3]. The anticipated need for surgical interventions, prolonged hospitalization, and ongoing medical care may also have significant implications for family life [4,5]. The psychological process underlying the decision to continue or terminate a pregnancy is itself shaped by a complex interplay of fetal, personal, and contextual factors, including hope, perceived quality of life, and the management of diagnostic uncertainty [6,7]. For this reason, prenatal counselling in CHD extends beyond anatomical description and requires a broader evaluation of prognosis. These broader clinical considerations may ultimately influence how parents approach the option of termination.

Previous studies have shown that factors such as cardiac severity, gestational age at diagnosis, genetic findings, and associated extracardiac anomalies may influence the likelihood of termination [8,9]. However, these factors are closely related to one another, making it difficult to determine their independent effects on parental decisions. Moreover, most available data on this topic originate from single-institution or national cohorts with heterogeneous counselling settings, where variations in institutional practice, physician perspective, and available services may influence how information is delivered and interpreted [10,11].

In many tertiary referral centers, multidisciplinary fetal councils aim to provide structured and consistent counselling by integrating cardiology, maternal–fetal medicine, genetics, and neonatology perspectives [12]. Understanding which clinical factors independently shape parental decisions within such a standardized framework may help refine counselling strategies and improve communication of risk and prognosis. However, few studies have examined parental decision-making specifically within a single, standardized multidisciplinary council model, where variability introduced by differing institutional practices and physician perspectives is minimized [10,11]. This represents an important gap, as most previous data are derived from heterogeneous settings that may confound the identification of true clinical determinants of parental choice.

The present study was therefore designed to evaluate the clinical determinants of parental acceptance of TOP following prenatal CHD diagnosis in cases where termination was formally recommended within a structured multidisciplinary setting.

## 2. Materials and Methods

### 2.1. Study Design

This retrospective cohort study was carried out at Izmir City Hospital, Turkey, a tertiary referral center where prenatal CHD cases are routinely evaluated within a structured multidisciplinary perinatology council. The study population consisted of pregnancies with a confirmed prenatal diagnosis of CHD that were discussed in the council and for which TOP was medically recommended between December 2023 and February 2026. This interval corresponds to the full period since the establishment of the hospital’s fetal cardiology and multidisciplinary perinatology council; as a newly opened tertiary referral center, no earlier institutional data were available for inclusion.

All cardiac diagnoses were established by detailed fetal echocardiography performed by experienced maternal–fetal medicine specialists in collaboration with pediatric cardiologists. Cases were subsequently presented to the multidisciplinary council, which includes specialists in perinatology, pediatric cardiology, neonatology, medical genetics, and pediatric cardiovascular surgery when required. Clinical information reviewed during council meetings included complete fetal echocardiographic findings, comprehensive anatomic survey results, genetic-evaluation status, and associated extracardiac anomalies.

After multidisciplinary assessment, a formal medical recommendation regarding pregnancy continuation or termination was documented. Although the council provided a structured medical opinion, the ultimate decision regarding acceptance or refusal of termination remained with the parents. Each case was initially presented to the council at the time of diagnosis and was re-evaluated whenever genetic results became available or when a significant change occurred in cardiac severity or fetal status. Parents were counselled separately by each relevant specialty both before the council meeting and again afterward when clinically indicated. Following the council’s deliberation, the recommendation was communicated to the parents, and their decision was documented with written informed consent. No formal published guideline defines fixed criteria for recommending termination versus continuation in prenatally diagnosed CHD; consistent with the approach described in other multidisciplinary council-based studies [12], the recommendation instead reflects a consensus clinical judgment reached collectively by all attending specialties, integrating cardiac severity, genetic and extracardiac findings, and overall prognosis for the individual case. When cardiac severity alone was judged sufficient to support a termination recommendation, the council issued that recommendation without awaiting genetic results. When the initial council did not reach a recommendation to terminate and the family requested genetic testing, the case was reassessed after results became available, typically after an interval of approximately three to four weeks (reflecting, in particular, the turnaround time for chromosomal microarray analysis); a termination recommendation could then be issued if the genetic result revealed an abnormality. All genetic results reported in this study were therefore available before the final parental decision. The legal gestational-age limit for termination in Turkey does not constrain council decision-making in this setting, since termination following a council recommendation is not subject to the same gestational limit that applies to elective termination on request; the council’s own recommendation, rather than the approaching legal window, determined the pace of the decision-making process.

Singleton pregnancies with a confirmed prenatal diagnosis of congenital heart disease, a documented TOP recommendation by the multidisciplinary fetal council, and maternal age ≥18 years were eligible for inclusion. Multiple gestations and cases with incomplete clinical or echocardiographic data preventing accurate classification of cardiac diagnosis, severity category, or parental decision were excluded.

### 2.2. Genetic Evaluation

Genetic counseling was routinely offered in collaboration with the medical genetics department. Counseling addressed the potential association between complex CHD and chromosomal or monogenic disorders, available prenatal diagnostic methods, and their limitations. When clinically indicated, invasive prenatal genetic testing was performed after informed parental consent, in accordance with institutional practice. Genetic testing followed a sequential protocol: conventional karyotyping was performed first, and testing proceeded to chromosomal microarray only if the karyotype was normal; whole-exome sequencing was pursued, at the family’s own expense through an external laboratory, only if both prior tests were normal and a monogenic etiology remained clinically suspected.

In cases with structural anomalies, conventional karyotyping and chromosomal microarray analysis were offered as first-line diagnostic tests. Whole-exome sequencing (WES) was considered in selected cases with normal karyotype and microarray results when a monogenic etiology was suspected; however, WES was not routinely performed at our institution and required referral to an external laboratory after separate parental consent. Karyotyping and chromosomal microarray analysis were performed at the institution’s own cytogenetics and molecular genetics laboratory using standard clinical protocols. Whole-exome sequencing is not covered by the national health insurance system in Turkey; when clinically indicated, it was arranged privately by the family through external commercial laboratories following separate informed consent. Genetic variants were classified according to the American College of Medical Genetics and Genomics (ACMG) criteria into pathogenic, likely pathogenic, variant of uncertain significance (VUS), likely benign, or benign categories. Chromosomal microarray analysis was performed on DNA extracted from peripheral blood using the Applied Biosystems CytoScan™ HT-CMA^®^ Assay, with data analyzed in Chromosome Analysis Suite (ChAS) software version 4.5 (Thermo Fisher Scientific, Waltham, MA, USA), against the GRCh38 (hg38) reference genome. Whole-exome sequencing was not performed through a single designated provider; families arranged testing privately, and the specific commercial laboratory and assay used therefore varied according to the family’s own arrangement.

### 2.3. Data Collection

Clinical and demographic data were retrospectively obtained from electronic medical records and council documentation. Maternal characteristics included age, gravidity, parity, number of living children, and history of previous abortion. Fetal variables comprised gestational age at diagnosis, primary cardiac diagnosis, cardiac severity category, presence of extracardiac structural anomalies, intrauterine growth restriction (IUGR), and genetic-evaluation status and results. Extracardiac anomalies were recorded according to the affected organ system. The hospital’s electronic medical record does not routinely capture sociodemographic variables such as parental educational level or income, and these were therefore not available for analysis. The council’s medical recommendation regarding pregnancy continuation or termination is recorded in the multidisciplinary council minutes and the electronic medical record for each case.

Gestational age at diagnosis was evaluated both as a continuous variable and as predefined clinical categories (<20 weeks, 20–23 + 6 weeks, and ≥24 weeks) to reflect relevant diagnostic intervals in prenatal practice. The primary endpoint of the study was parental acceptance of the recommended termination of pregnancy.

### 2.4. Cardiac Classification

Each fetus was assigned a single primary cardiac diagnosis based on detailed prenatal echocardiography. Cardiac severity was evaluated using the Fetal Cardiovascular Disease Severity Scale described by Davey et al. [13]. This scale classifies structural congenital heart disease into seven levels (grade 1–7) according to anatomical complexity, anticipated postnatal intervention, the likelihood of biventricular repair versus single-ventricle palliation, and overall expected prognosis. Extracardiac or genetic findings are not included in the scoring system and are considered separately.

For the purposes of statistical analysis, the original seven grades were grouped into three categories as low (grades 1–2), moderate (grades 3–4), and high severity (grades 5–7). This grouping was used for analytical purposes and to reflect routine clinical counseling practice, where severity is typically communicated in broader categories rather than individual numeric grades.

### 2.5. Statistical Analysis

All statistical analyses were performed using IBM SPSS Statistics version 26.0. Distribution of continuous variables was assessed using the Kolmogorov–Smirnov test. As none of the continuous variables showed a normal distribution, they are presented as median (interquartile range, IQR). Categorical variables are expressed as number and percentage.

Comparisons between cases in which termination was accepted and those in which it was refused were conducted using the Mann–Whitney U test for continuous variables and the chi-square or Fisher’s exact test for categorical variables, as appropriate.

To determine variables independently associated with parental acceptance of termination, binary logistic regression analysis was performed using a simplified, clinically prespecified model: gestational age at diagnosis (continuous), cardiac severity (severe vs. low/moderate), and genetic-evaluation status (not performed, major chromosomal/pathogenic anomaly, or normal). Extracardiac anomaly classification was not included in the multivariable model and is reported descriptively. Odds ratios (ORs) and 95% confidence intervals (CIs) were calculated. Model adequacy was evaluated using −2 log likelihood, Cox & Snell and Nagelkerke pseudo-R^2^ statistics, and the Hosmer–Lemeshow goodness-of-fit test. A subgroup analysis was additionally performed in fetuses with isolated CHD, defined as absence of extracardiac structural anomalies. Statistical significance was defined as a two-sided *p* value < 0.05. For categorical variables with more than two levels in Table 1 and Table 2, an overall test of association is reported rather than separate category-by-category comparisons.

### 2.6. Ethical Considerations

The study protocol was approved by the local non-interventional clinical research ethics committee (Approval No: 2026/107). Owing to the retrospective nature of the study, informed consent was waived. All data were anonymized prior to analysis, and the study was conducted in accordance with the principles of the Declaration of Helsinki.

## 3. Results

A total of 62 pregnancies with a prenatal diagnosis of CHD, for which termination of pregnancy was recommended by the multidisciplinary council, were included in the analysis. Of these, the parents of 41 pregnancies (66.1%) decided to terminate the pregnancy, whereas 21 (33.9%) decided to continue it.

Maternal age, gravida, parity, and number of living children were similar between the groups (all *p* > 0.05). The median maternal age was 29 years in both groups. Primigravidity did not differ significantly between groups (34.1% vs. 28.6%, *p* = 0.777). Previous abortion history was not associated with the decision (26.8% vs. 23.8%, *p* = 1.000). Detailed maternal and obstetric characteristics are presented in Table 1.

The median gestational age at diagnosis was comparable between the groups (22 vs. 21 weeks, *p* = 0.521). When stratified into gestational age categories (<20 weeks, 20–23 + 6 weeks, ≥24 weeks), no statistically significant overall association with the decision was observed (*p* = 0.906).

As shown in Table 2, termination rates varied numerically according to cardiac severity: 60.0% of cases with low-complexity CHD, 75.0% of cases with moderate-complexity CHD, and 63.9% of cases with high-complexity CHD ended in a parental decision to terminate, but this variation was not statistically significant overall (*p* = 0.667). Extracardiac anomaly classification was likewise not significantly associated with the decision overall (*p* = 0.855). Genetic findings, in contrast, showed a significant overall association with the decision (*p* = 0.019): the parents of 81.0% of cases with a major chromosomal or pathogenic anomaly and 80.0% of cases with a normal genetic-evaluation status decided to terminate, compared with 46.2% of cases in which genetic testing was not performed.

A simplified multivariable logistic regression model, comprising three clinically prespecified variables (gestational age at diagnosis, cardiac severity, and genetic-evaluation status; four parameters in total), was fitted to identify variables independently associated with parental acceptance of termination (Table 3). Genetic-evaluation status was the only variable independently associated with termination acceptance. Compared with cases in which genetic testing was not performed, the presence of a major chromosomal or pathogenic anomaly was associated with increased odds of termination (adjusted OR 4.97; 95% CI 1.29–19.11; *p* = 0.020). Similarly, cases with a normal genetic-evaluation status also showed increased odds of termination (adjusted OR 4.71; 95% CI 1.07–20.77; *p* = 0.041). Gestational age (adjusted OR 0.96; 95% CI 0.85–1.09; *p* = 0.562) and cardiac severity (adjusted OR 1.17; 95% CI 0.37–3.73; *p* = 0.789) were not independently associated with the termination decision. The model demonstrated a Nagelkerke R^2^ of 0.176 (Cox & Snell R^2^ 0.127) with a −2 log likelihood value of 70.94, and the Hosmer–Lemeshow goodness-of-fit test yielded *p* = 0.120; given the limited power of this test in small samples, this result should not be taken as confirmation of model adequacy, although it did not indicate evidence of lack of fit.

**Table 1:** Comparison of Maternal and Clinical Characteristics According to Termination Decision.

Variable	Pregnancy Continued (*n* = 21)	Termination Decision (*n* = 41)	*p* Value
Maternal age (years), median (IQR)	29 (24–34)	29 (25–33)	0.602
Gravida, median (IQR)	2 (1–3)	2 (1–3)	0.512
Parity, median (IQR)	1 (0–2)	1 (0–2)	0.752
Living children, median (IQR)	1 (0–2)	1 (0–2)	0.819
Previous abortion history, *n* (%)	5 (23.8)	11 (26.8)	1.000
Primigravida, *n* (%)	6 (28.6)	14 (34.1)	0.777
GA at diagnosis (weeks), median (IQR)	22 (19–26)	21 (19–25)	0.521
GA category, *n* (%)			0.906
<20 weeks	6 (35.3)	11 (64.7)	
20–23 + 6 weeks	7 (30.4)	16 (69.6)	
≥24 weeks	8 (36.4)	14 (63.6)	

Continuous variables are presented as median (interquartile range, IQR) and were compared using the Mann–Whitney U test. Categorical variables are presented as *n* (%) and were compared using the chi-square or Fisher’s exact test as appropriate. Abbreviations: GA: gestational age.

**Table 2:** Fetal Clinical Characteristics According to Termination Decision.

Variable	Continued (*n* = 21) *n* (%)	Terminated (*n* = 41) *n* (%)	Total	*p* Value
Cardiac Severity (3-category)				0.667
Low	4 (40.0)	6 (60.0)	10	
Moderate	4 (25.0)	12 (75.0)	16	
Severe	13 (36.1)	23 (63.9)	36	
Extracardiac Anomaly Classification				0.855
None	10 (43.5)	13 (56.5)	23	
Hydrops/cystic hygroma	2 (33.3)	4 (66.7)	6	
Skeletal/extremity anomalies	2 (20.0)	8 (80.0)	10	
Multiple malformations/syndromic	3 (42.9)	4 (57.1)	7	
Renal/GIS anomalies	1 (25.0)	3 (75.0)	4	
Soft marker only	1 (25.0)	3 (75.0)	4	
CNS anomalies	2 (25.0)	6 (75.0)	8	
Genetic-Evaluation Status				0.019
Not performed	14 (53.8)	12 (46.2)	26	
Major chromosomal/pathogenic anomaly	4 (19.0)	17 (81.0)	21	
Normal	3 (20.0)	12 (80.0)	15	

Values are presented as *n* (%). *p* values were calculated for each category using the chi-square or Fisher’s exact test as appropriate. Abbreviations: GIS, gastrointestinal system; CNS, central nervous system.

**Table 3:** Multivariable Logistic Regression Analysis for Factors Associated with Termination Acceptance.

Variable	Adjusted OR	95% CI	*p* Value
Gestational age at diagnosis (per week increase)	0.96	0.85–1.09	0.562
Cardiac severity: low/moderate (vs. severe)	1.17	0.37–3.73	0.789
Genetic-evaluation status: major chromosomal/pathogenic anomaly (vs. not performed)	4.97	1.29–19.11	0.020
Genetic-evaluation status: normal (vs. not performed)	4.71	1.07–20.77	0.041

Reference categories: ≥24 weeks for gestational age category; severe CHD for cardiac severity; not performed for genetic-evaluation status; no extracardiac anomaly for anomaly classification. OR: odds ratio; CI: confidence interval; CHD: congenital heart disease.

## 4. Discussion

In pregnancies complicated by a prenatal diagnosis of CHD, the decision regarding TOP is often shaped by multiple clinical and prognostic factors. In this retrospective cohort study conducted within a structured multidisciplinary fetal council, we investigated the clinical determinants of parental acceptance of TOP when termination was medically recommended. Among the 62 cases in which termination was medically recommended, approximately two-thirds of parents accepted termination. In multivariable analysis, the only variable independently associated with acceptance of termination was the genetic-evaluation status. Gestational age at diagnosis, cardiac severity and extracardiac anomalies were not identified as independent predictors.

The influence of genetic evaluation on parental decision-making during prenatal counselling is well recognized in the literature [14]. Several studies have shown that the presence of a major chromosomal or pathogenic anomaly significantly increases termination rates [15,16]. In a cohort of 1035 fetuses with CHD reported by Zhang et al., the termination rate was 93.1% in cases with a chromosomal anomaly, compared with 15.5% in fetuses with a normal CMA result [16]. Similarly, Chih et al. identified chromosomal/genetic anomalies as a strong predictor of termination [15]. Bensemlali et al. also demonstrated that the presence of a cytogenetic anomaly or major extracardiac malformation increased the likelihood of choosing TOP [17]. These associations, like ours, are drawn from observational cohorts in which the temporal and causal relationship between testing and the decision cannot be fully disentangled.

In our simplified model, major/pathogenic genetic anomalies were associated with acceptance of termination (aOR 4.97; 95% CI 1.29–19.11; *p* = 0.020). A normal genetic-evaluation status was also associated with increased odds of termination (aOR 4.71; 95% CI 1.07–20.77; *p* = 0.041). It should be noted that this ‘normal’ category is not biologically homogeneous, as detailed below, since it spans different depths of testing (karyotype alone, karyotype plus chromosomal microarray, and, in a minority of cases, whole-exome sequencing). This finding suggests that being involved in the genetic evaluation process itself, regardless of the specific result, may be associated with parental decision-making. Cases referred for genetic testing may represent clinically more complex or prognostically uncertain situations. Therefore, undergoing genetic testing may reflect not only biological findings but also a higher level of clinical concern during counselling. This pattern may also reflect confounding by indication, in which the clinical suspicion that prompted genetic testing, rather than the test itself, is associated with the decision-making process; the counselling encounters associated with invasive testing may further increase parental engagement with the perceived severity of the diagnosis, independent of the eventual result. Because families with more complex cases may have been more likely to be offered or to accept testing, because families choosing testing may differ in unmeasured psychosocial characteristics, and because the timing of testing and of the legal decision-making window may be interdependent, genetic-evaluation status should be interpreted as a variable associated with the decision to terminate rather than as a causal determinant of it. From a clinical perspective, this finding suggests that counselling should explicitly address that a normal genetic-evaluation status does not exclude all genetic etiologies and should not be interpreted as altering the prognosis associated with the cardiac defect itself. In our cohort, karyotyping and chromosomal microarray were performed sequentially, with whole-exome sequencing pursued privately by the family only when both prior tests were normal; the ‘normal’ genetic-evaluation status category in Table 2 may therefore include cases tested to different depths, which we were unable to fully disaggregate and note as a limitation.

Regarding cardiac severity, although higher termination rates were observed in the moderately complex CHD group in univariable analysis, this association did not remain significant when severity was modeled as a binary variable (severe vs. low/moderate; aOR 1.17; 95% CI 0.37–3.73; *p* = 0.789) in the simplified multivariable model. In a cohort of 73 cases reported by Nakao et al., the termination rate in the complex CHD group was 44%, but this variable was not significant in multivariable analysis (aOR 0.56; 95% CI 0.14–2.20) [18]. In contrast, the critical CHD group showed a termination rate of 77% and was identified as an independent predictor (aOR 5.25; 95% CI 1.09–25.19). In the larger cohort reported by Verbeke et al., an association between cardiac severity and termination was observed; however, a clear linear dose–response pattern across severity categories was not demonstrated, and statistical significance was lost in some intermediate groups in multivariable analysis (*p* > 0.05) [19]. These findings suggest that the impact of cardiac severity may depend on how categories are defined, underlying prognostic heterogeneity, and accompanying clinical factors.

Gestational age at diagnosis was not independently associated with termination in our simplified multivariable analysis (aOR 0.96; 95% CI 0.85–1.09; *p* = 0.562). Extracardiac anomaly classification could not be evaluated in the multivariable model because its seven categories were too sparse relative to our sample size, and is therefore reported descriptively in Table 2, where none of its categories showed a significant univariable association with the outcome. Although early diagnosis has been reported to increase the likelihood of termination (e.g., Nakao et al.; aOR 0.94; 95% CI 0.89–0.99), and extracardiac anomalies have been shown to influence decision-making in other cohorts, these factors are often closely related to genetic findings and overall prognosis [18]. In the study by Perez et al., early fetal echocardiographic diagnosis and a higher fetal disease severity score were independently associated with termination, whereas extracardiac anomalies influenced decision-making mainly as components of the severity score rather than as isolated variables [20]. In our cohort, the lack of independent association for gestational age and cardiac severity may be explained by the dominant association of genetic-evaluation status and the fact that all cases were managed within a structured multidisciplinary counselling framework.

This study has several limitations. The single-center and retrospective nature of the study may limit the generalizability of the findings and should be considered when interpreting the results. The relatively small sample size may have resulted in wide confidence intervals and limited statistical power, particularly in subgroup analyses. In addition, psychosocial determinants such as parental education level, socioeconomic status, religious beliefs, and cultural values were not assessed, although these factors may contribute to decision-making. These factors have been reported to meaningfully shape parental decision-making after a severe prenatal diagnosis, and their absence from the present dataset represents an important unmeasured confounder that the retrospective, single-center design did not allow us to capture systematically. Furthermore, legal and counselling frameworks governing TOP vary substantially across countries. In Turkey, pregnancy may be terminated upon maternal request only up to 10 weeks of gestation; beyond this limit, termination is permitted solely following a formal recommendation by an authorized medical council. Consequently, our findings, obtained within this specific legal and institutional context, may not be directly generalizable to healthcare systems with different legislative frameworks or counselling practices. Because only pregnancies for which the council had already recommended termination were included, the cohort is highly selected and excludes fetuses with CHD for whom termination was not recommended; the findings therefore describe parental decision-making within a group already judged eligible for or advised to undergo termination, not parental decision-making after prenatal CHD diagnosis in general. Because the same clinical features evaluated here as candidate predictors may also have influenced the council’s initial recommendation, conditioning inclusion on that recommendation may introduce selection or collider bias, and this should be considered when interpreting the associations reported [21].

Despite these limitations, the study also has important strengths. All cases were evaluated within a structured multidisciplinary fetal council, ensuring a consistent and comprehensive, though not necessarily standardized, counselling process. Furthermore, inclusion was limited to cases in which termination was formally recommended, allowing analysis within a clinically meaningful population. Finally, evaluating genetic findings, cardiac severity, and gestational age together in a multivariable model allowed for a clearer assessment of their independent effects.

These findings carry several practical implications for clinical counselling. Because genetic-evaluation status, rather than the specific result, was most strongly associated with parental decision-making, counselling teams should ensure that families receive equally clear support whether or not invasive testing is pursued, and should explicitly address that a normal genetic-evaluation status does not by itself indicate a more favorable prognosis. The apparent centrality of genetic-evaluation status in this cohort also suggests that access to genetic counselling and testing should be prioritized and, where possible, standardized across centers, since disparities in access, such as out-of-pocket cost for whole-exome sequencing, could otherwise translate into disparities in the information available to guide parental decisions. Because all cases in this cohort were managed within a single multidisciplinary council and no comparator group without council involvement was available, this study cannot determine whether the council itself improved counselling quality or changed parental decisions; comparative studies against non-council-based care would be needed before recommending wider adoption on this basis. Future research should prioritize multicenter, adequately powered studies that incorporate psychosocial and socioeconomic variables alongside clinical factors, in order to build more comprehensive, generalizable models of parental decision-making after a prenatal CHD diagnosis.

In conclusion, in this selected cohort of pregnancies with a prenatal diagnosis of CHD for which termination was recommended, genetic-evaluation status showed the strongest statistical association with the decision within a simplified, four-parameter multivariable model constrained by a limited sample size, and these findings should be interpreted with due caution given the wide confidence intervals observed. Although cardiac severity and gestational age showed trends in univariable analyses, they were not identified as independent predictors. Larger, multicenter studies incorporating psychosocial variables are needed to confirm these findings and to better understand the complex structure of parental decision-making in this setting.

## Data Availability

The data presented in this study are available on request from the corresponding author due to privacy and ethical restrictions.

## Abbreviations

The following abbreviations are used in this manuscript:

CHD	Congenital heart disease
TOP	Termination of pregnancy
GA	Gestational age
IUGR	Intrauterine growth restriction
WES	Whole-exome sequencing
CMA	Chromosomal microarray analysis
OR	Odds ratio
aOR	Adjusted odds ratio
CI	Confidence interval
GIS	Gastrointestinal system
CNS	Central nervous system
